# Pulpal Thermal Changes following Er-YAG Laser Debonding of Ceramic Brackets

**DOI:** 10.1155/2014/912429

**Published:** 2014-08-12

**Authors:** Didem Nalbantgil, Murat Tozlu, Mehmet Oguz Oztoprak

**Affiliations:** ^1^Department of Orthodontics, Faculty of Dentistry, Yeditepe University, 34730 Istanbul, Turkey; ^2^Private Practice, 34730 Istanbul, Turkey

## Abstract

Lasers are effective in debonding ceramic brackets. Unfortunately, while reducing the adhesive bond strength, lasers are also reported to increase pulpal temperature. The aim of this study was to evaluate the shear bond strengths and temperature increase levels after debonding ceramic brackets using an Er-YAG laser with or without water-cooling. Sixty polycrystalline upper premolar ceramic brackets were placed on the labial surface of sixty human premolar teeth which were randomly divided into three groups of twenty. A laser pulse at 5 W for 9 seconds was delivered to each bracket in both study groups either with water-cooling (water group) or without water-cooling (waterless group) using an Er-YAG laser. Debonding was performed 45 seconds after laser exposure and shear bond strengths were measured. Data comparison revealed a statistically significant difference between the groups. Mean temperature increases of 2.41°C and 4.59°C were recorded for the water and waterless laser groups, respectively. The shear bond strength value for the control group was 22.76 MPa and 10.46 and 6.36 MPa for the water and waterless laser groups, respectively. The application of Er-YAG laser with water-cooling was an efficient and safe method of debonding ceramic brackets.

## 1. Introduction

The use of lasers eliminates problems such as enamel tears, bracket failure, and pain encountered during the debonding of ceramic brackets [1–3]. Laser use significantly decreases the force needed to debond brackets by thermal softening of the adhesive resin [4–7] and therefore provides a mechanism for the safe removal of the ceramic brackets from the enamel surface. Previous literature has reported variables like different laser types, bracket types, resin composition, and application methods, in an attempt to determine optimal laser parameters [1, 8–11]. Additional studies have investigated laser application duration and energies in order to assess iatrogenic damage to pulpal tissue [2, 9–12]. As a benchmark, previous laser debonding studies used a safety threshold of a 5.5°C increase in intrapulpal temperature after which 85 percent of teeth remained vital at this level of temperature increase. It was further determined that there were no adverse pulpal effects with an intrapulpal temperature increase of 1.8°C [13].

In many debonding studies which used Nd:YAG or CO_2_ lasers, the bracket debonding force was applied immediately after or during lasing [8, 10, 11]. Clinically, this method created a risk of dropping a hot bracket into an oral cavity and the need to use extra equipment to secure the brackets. It would be advantageous if the clinician had a simple and easy method that allows the debonding of ceramic brackets in a similar fashion to traditional debonding techniques.

It has been reported that Er-YAG lasers have a lesser thermal effect than Nd-YAG or CO_2_ lasers [14]. In addition, Er-YAG laser has been used to etch tooth surfaces [15–18] and to remove residual composite resin after bracket debonding [19, 20]. Oztoprak et al. [6] reported the effectiveness of Er-YAG laser on debonding of orthodontic ceramic brackets using a scanning method. The method was defined as the thorough scanning of the bracket surface with horizontal movements parallel to bracket slot with an application tip positioned perpendicularly 2 mm from the bracket. Scanning through the bracket and debonding 45 seconds after laser exposure were shown to be more practical than immediate shearing. It was concluded that, with the aid of scanning techniques, ceramic brackets could be debonded like conventional metal brackets without the involvement of extra equipment or procedures. However, Oztoprak et al. [6] failed to report changes in pulpal temperature. Therefore the aim of the current study was to determine the pulpal temperature changes using an Er-YAG laser employing the scanning method either with or without water-cooling. A secondary aim was to evaluate and compare the shear bond strengths required for bracket removal.

## 2. Material and Methods

**Figure 1:**
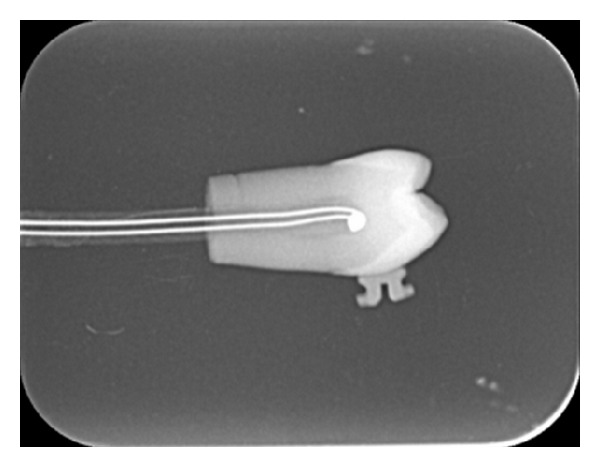
Radiographic image of the thermocouple positioned inside the pulp.

Sixty freshly extracted human premolar teeth were randomly divided into three groups of twenty. Sixty polycrystalline upper premolar ceramic brackets (Transcend, 3M Unitek, Monrovia, CA, USA) were placed on the buccal surface of the teeth using Transbond XT (3M Unitek, Monrovia, CA, USA) as the orthodontic composite adhesive after tooth surface conditioning with 37 percent phosphoric acid for 15 seconds. The composite resin was light cured for 20 seconds with a halogen light curing unit (Optilux, Kerr, Orange, CA, USA). The first group was assigned as the control group and no laser application was performed. The other two groups were assigned as test groups and the pulpal tissues of these teeth were removed with an endodontic file to facilitate the placement of a thermocouple. A 0.2 mm diameter K-type thermocouple (Ishifuku Metal Industry, Tokyo, Japan) was positioned so that its sensor contacted the surface of the pulp dentinal wall, directly under the bracket. The position of the thermocouple was verified radiographically (Figure 1). The thermocouple was calibrated and the room temperature set at 25°C. The pulpal temperature change was continuously monitored (XY Recorder WX2400, Graphtec Corp., Tokyo, Japan) and before testing, all samples were stored in distilled water at 37°C for 48 hours.

**Figure 2:**
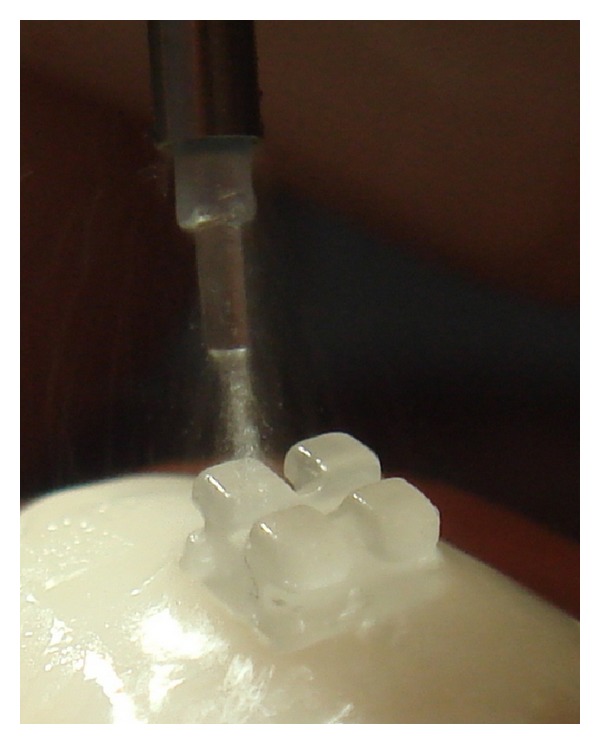
Application of the laser beam.

The Er-YAG laser (DEKA Smart 2940 D Plus, VersaWave, Hoya Conbio, Fremont, CA, USA) at a power of 5 W with a wavelength of 2940 nm was used for this study. Laser energy was applied on the surface of the brackets for 9 seconds by scanning the surface of the bracket (Figure 2). The application tip, of 1 mm diameter, was positioned perpendicularly 2 mm from each bracket [6] and a pulse was delivered to all teeth at 5 W for 9 seconds with water-cooling (water group) or without water-cooling (waterless group). The force required to debond the brackets was applied 45 seconds after laser exposure [6] and shear bond strengths were measured in megapascals (MPa) at a crosshead speed of 1 mm/minute by an Instron universal testing machine.

Statistical calculations were performed with GraphPad Prism V.3 software for Windows. In addition to standard descriptive statistical calculations (mean and standard deviation), one way ANOVA was used for group comparison, and a post hoc Tukey multiple comparison test was performed to identify differences. An unpaired *t*-test was used for the comparison of the intrapulpal temperatures between the groups. The statistical significance level was established at  *P* < 0.05.

## 3. Results

The results revealed statistically significant differences between the control, water, and the waterless groups (*P* < 0.05). The mean shear bond strength was 22.76 MPa for the control group, 10.46 MPa for the water-cooled group, and 6.36 MPa for the waterless group, respectively (Table 1). Also, the post hoc Tukey comparison test revealed significant differences for the shear bond strengths between the three groups (Table 2). A statistically significant difference was also seen in the mean temperature increases between the groups (*P* < 0.05). The mean increases were 2.41°C and 4.59°C with standard deviations of 0.25°C and 0.48°C for the water and waterless laser groups, respectively (Table 3).

## 4. Discussion

Animal experiments have shown that the circulation in the pulp tissue is altered by a 3°C temperature increase [21]. Hyperaemia results from vasodilation following a heat-induced elevation in temperature from 37°C to 39°C [21]. Zach and Cohen [13], in an* in vivo* study, demonstrated that an increase in pulpal temperature to 42.2°C caused pulpal necrosis in 15 percent of the teeth in a Macaca sample. A rise in temperature to 47.7°C caused necrosis in 60 percent of the teeth generating the conclusion that pulp tissue was highly susceptible to thermal stress. The thermal effects of laser debonding of ceramic brackets were justified and evaluated to determine the safest and most suitable method.

In the current study, an Er-YAG laser was chosen because of its reported reduced thermal effect compared with a Nd:YAG or a CO_2_ laser [14, 22]. In addition, an Er-YAG laser emits a wavelength of 2904 nm which corresponds to the main absorption peak of water [23]. Thus, an Er-YAG laser may be highly absorbed by the adhesive bonding resin containing water or residual monomer.

It was unlikely that the monitoring of pulp temperature in the current study replicated the* in vivo* condition since water was absent from the dentinal tubules. This likely affected tooth thermal conductivity and presented the pulp to the full effects of the temperature change. It is further likely that any thermal increase is faster and higher when water is absent from the environment and the intrapulpal temperature increase would expectedly be less in the* in vivo* situation.

The current study revealed a statistically significant difference in the shear bond strength in the debonding force delivered to the control group and the laser groups. This result is consistent with the previous studies [4, 5]. In addition, there was a statistical significance in shear bond strength required to debond brackets in the water and waterless laser groups in addition to a statistically significant intrapulpal temperature change between the two groups. Less than half the intrapulpal temperature increase was observed with water-cooling when compared with the laser group without water-cooling. However, both water and waterless groups displayed acceptable shear bond strengths [24]. Furthermore, intrapulpal temperature increases for both groups were within the safety limits suggested by previously published critical values for pulp survival. Since the waterless group presented findings approaching these critical values, the water group appeared to be safer and still reliable in reducing shear bond strength and controlling intrapulpal temperature increase.

## 5. Conclusion

Er-YAG laser irradiation and water-cooling with the scanning application method produced the following findings.

1. Er-YAG laser-aided debonding, with or without water-cooling, was effective for debonding ceramic brackets by reducing resin shear bond strength.
2. Er-YAG laser application with water-cooling appeared to be a safer option by reducing resin shear bond strength and reducing the likelihood of intrapulpal temperature increase while debonding ceramic brackets.

## Figures and Tables

**Table 1:** Comparison of the mean data for the shear bond strengths and the standard deviations of the groups.

	Control group	Waterless group	Water group	*P*
Shear bond strength (in MPa)	22.76 ± 2.99	6.36 ± 1.92	10.46 ± 2.25	∗∗∗

Statistically significant (***P < 0.05) changes.

±: standard deviation.

**Table 2:** Intergroup comparison of the shear bond strengths of the groups.

Tukey's multiple comparison test	Shear bond strength
Control/water group	∗∗∗
Control/waterless group	∗∗∗
Waterless group/water group	∗∗∗

Statistically significant (***P < 0.05) changes.

**Table 3:** Comparison of mean increase in intra-pulpal temperature for the water and waterless groups.

	Waterless group	Water group	*P*
Increase in intra-pulpal temperature in °C	4.59 ± 0.48	2.41 ± 0.25	∗∗∗

Statistically significant (***P < 0.05) changes.
